# Genome-Wide Identification and Characterization of Glycosyltransferase Family 47 in Cotton

**DOI:** 10.3389/fgene.2019.00824

**Published:** 2019-09-11

**Authors:** Aimin Wu, Pengbo Hao, Hengling Wei, Huiru Sun, Shuaishuai Cheng, Pengyun Chen, Qiang Ma, Lijiao Gu, Meng Zhang, Hantao Wang, Shuxun Yu

**Affiliations:** ^1^State Key Laboratory of Cotton Biology, Institute of Cotton Research, Chinese Academy of Agricultural Sciences, Anyang, China; ^2^National Key Laboratory of Crop Genetic Improvement, Huazhong Agricultural University, Wuhan, China

**Keywords:** *Gossypium*, glycosyltransferase, gene expansion, fiber development, stress response

## Abstract

The glycosyltransferase (GT) 47 family is involved in the biosynthesis of xylose, pectin and xyloglucan and plays a significant role in maintaining the normal morphology of the plant cell wall. However, the functions of GT47s are less well known in cotton. In the present study, a total of 53, 53, 105 and 109 GT47 genes were detected by genome-wide identification in *Gossypium arboreum*, *G. raimondii*, *G. hirsutum* and *G. barbadense*, respectively. All the GT47s were classified into six major groups *via* phylogenetic analysis. The exon/intron structure and protein motifs indicated that each branch of the GT47 genes was highly conserved. Collinearity analysis showed that GT47 gene family expansion occurred in *Gossypium spp*. mainly through whole-genome duplication and that segmental duplication mainly promoted GT47 gene expansion within the A and D subgenomes. The Ka/Ks values suggested that the GT47 gene family has undergone purifying selection during the long-term evolutionary process. Transcriptomic data and qRT-PCR showed that GhGT47 genes exhibited different expression patterns in each tissue and during fiber development. Our results suggest that some genes in the GhGT47 family might be associated with fiber development and the abiotic stress response, which could promote further research involving functional analysis of GT47 genes in cotton.

## Introduction

Glycosyltransferases (GTs) (EC 2.4.x.y) are responsible for the transport of active sugars from donor molecules to acceptor molecules, forming glycosidic bonds (Sinnott, 1990; Lairson et al., 2008). According to gene production and sequence similarity, a total of 105 distinct GT subfamilies were identified in the Carbohydrate-Active Enzymes (CAZy) database (CAZypedia Consortium, 2018). Many GT subfamilies have been identified in different species. GT1 genes have been found in dicotyledons and monocotyledons, for example, maize (Li et al., 2014b), soybean (Mamoon Rehman et al., 2016), *Arabidopsis* (Li et al., 2001), flax (Barvkar et al., 2012) and cotton (Huang et al., 2015); GT8 members have been identified in 15 plant species (Yin et al., 2010); and GT43 members have been identified in charophycean green algae (Taujale and Yin, 2015), cotton (Li et al., 2014b) and rice (Lee et al., 2014), among others. Many GT gene families (GT2s, GT8s, GT31s, GT34s, GT37s and GT47s) have been suggested to be involved in cell wall component synthesis (cellulose, hemicellulose and pectin) and are listed on the cell wall genomics website (https://cellwall.genomics.purdue.edu/families/index.html).

The GT47 family was identified in plants according to the β-glucuronyltransferase domain (pf03016) (Zhong and Ye, 2003). GT47 has xyloglucan β-galactosyltransferase (EC 2.4.1.-) and arabinan α-L-arabinosyltransferase (EC 2.4.2.-) biological activities in plants and is involved in the biosynthesis of xylan, xyloglucan, pectin and other polysaccharides (Zhong and Ye, 2003; Harholt et al., 2006). In *Arabidopsis*, 39 GT47s were identified and divided into six subgroups by phylogenetic analysis (Li et al., 2004). The functions of many GT47 genes have been verified.

*AtMUR3*, a representative gene of the GT47 gene family, encodes a xyloglucan galactosyltransferase that specifically catalyzes the XXXG core structure of xyloglucan to XXLG subunits (L side chain) by transporting the Gal residue to the third xylose residue (Li et al., 2004, 3). The mutant *mur3-3* showed a defect in cell elongation and was characterized by wrinkled leaves, short petioles and a short hypocotyl in *Arabidopsis* (Tamura et al., 2005). The *AtMUR3* gene can maintain the stability of endomembranes by promoting the interaction between actin ﬁlaments and Golgi stacks in *Arabidopsis* (Tamura et al., 2005).

Another gene, *ARABINAN DEFICIENT1* (*ARAD1*), has been suggested to participate in the biosynthesis of rhamnogalacturonan I (RGI) (Harholt et al., 2006; Bethke et al., 2016), thereby increasing the content of pectin polysaccharides and contributing to primary wall function with regard to cell strength, cell adhesion, stomatal function, and the defense response (Caffall and Mohnen, 2009). The mutation of *arad1* causes a speciﬁc reduction in arabinan content and may have pleiotropic effects (Harholt et al., 2006). *XYLOGALACTURONAN DEFICIENT1* (*XGD1*) is also involved in pectin biosynthesis. *XGD1* exhibits xylogalacturonan (XGA) activity and is involved in the biosynthesis of XGA (Willats et al., 2001; Harholt et al., 2010).

*Irregular Xylem IRX10* and *IRX10L* were identified as xylan xylosyltransferases, which together with two members of GT43, namely, *IRX9* and *IRX14*, are involved in xylan synthesis (Brown et al., 2009, 10). Xylan is the major component of hemicellulose in the secondary cell wall of dicotyledonous plants; for example, the *Arabidopsis* mutant *irx10* shows xylem abnormalities (Brown et al., 2009, 10). In rice, *OsGT47A*, the homologous gene of *AtIRX10*, exhibits functional conservation and is most likely involved in xylan synthesis (Zhang et al., 2014). In wheat, RNA interference (RNAi) silencing of the *AtIRX10* ortholog *TaGT47_2* decreases the arabinoxylan content in transgenic lines (Lovegrove et al., 2013).

Other GT47 genes have been verified to have biological functions; for example, *Fragile Fiber8 FRA8/IRX7* and *F8H/IRX7L* are involved in xylan synthesis (Zhong, 2005; Pena et al., 2007; Lee et al., 2009). In addition, the functions are redundant with the corresponding paralogous genes *IRX9-like* (*IRX9-L*), *IRX10-L* and *IRX14-L* (Wu et al., 2010). Additionally, GT47s play important roles in biological stress and abiotic stress responses. Pectin biosynthesis is critical for immunity in *Arabidopsis thaliana*, and soluble pectin can be released when plants interact with pathogens to induce an immune response (Bethke et al., 2016). In maize, 3 intron-poor GT47 genes (*Zm00001d048389*, *Zm00001d038905* and *Zm00001d042279*) have been identified that might be involved in the drought stress response (Tan et al., 2018).

Cotton is an important cash crop, providing more than 90% of the world’s lint production. At present, there are 45 diploid (2n = 2x = 26) species and five tetraploid (2n = 4x = 52) species in the cotton genus (Wendel and Cronn, 2003). Because of their economic value, *G. hirsutum* and *G. barbadense* have been widely planted worldwide. In terms of the evolutionary origin of allopolyploid cotton (*G. hirsutum* and *G. barbadense*), the A and D subgenomes of allopolyploid cotton likely derived from the ancestral isolated diploid genomes *G. arboreum* (AA, 2n = 2x = 26) and *G. raimondii* (DD, 2n = 2x = 26), respectively, approximately 1-2 million years ago (MYA), two diploid cotton species that were hybridized *via* transoceanic dispersal (Wendel and Cronn, 2003). Recent research shows that the allotetraploid formed approximately 1.7–1.9 MYA (Hu et al., 2019). These two diploid ancestor species and two widely cultivated tetraploid species are widely used to study the evolutionary and biological characteristics of cotton.

As the most important natural textile fiber, cotton is the main raw material of the textile industry. The allotetraploid cotton species (*G. hirsutum* and *G. barbadense*) are widely cultivated because of their high yield and excellent fiber quality. The cotton fiber is a single cell produced by the elongation and thickening of the ovule epidermis cell and is also a powerful single-cell model for the study of cell wall synthesis (Haigler et al., 2012). Previous studies in *Arabidopsis* and rice have shown the importance of GT gene families in cell wall component synthesis (Pauly and Keegstra, 2016). However, only a few studies have reported the roles of the GT gene in the cell wall synthesis of cotton. The GT1 gene family remains undefined in cotton, and *GhUGT73AC4, GhUGT73P1* and *GhUGT96A2* may be essential for fiber initiation (Huang et al., 2015). Overexpressed *GhGT43A1* and *GhGT43C1*, the homologous genes of *IRX9* and *IRX14*, partially rescue the *Arabidopsis* mutant *irx9* and *irx14* phenotypes to the wild-type level and increase their xylan content (Li et al., 2014a). The roles of the GT47 family in cotton are unknown. Therefore, it is significant and meaningful to identify and analyze the GT47 gene family in different cotton species, which can help elucidate the distribution and evolutionary relationships of the GT47 gene family in different cotton plants. In the present study, we performed a comprehensive analysis of the GT47 gene family in four cotton species, specifically two diploid cottons (*G. arboreum*, *G. raimondii*) and two allotetraploid cottons (*G. hirsutum*, *G. barbadense*). Our purpose was to explore the biological function of GT47s in the growth and development of cotton and cotton fiber. The main objectives of our study were to explore the phylogenetic relationships, gene structures, expansion patterns, cis-acting elements and expression patterns in the GT47 family. The present results help elucidate the potential functions of GhGT47 family genes and provide candidate genes for improving fiber quality and the stress response.

## Materials and Methods

### Identification of the GT47 Family in *Gossypium Spp*.

The genomes of *G. arboreum* (CRI, version 1.0), *G. raimondii* (JGI, version 2.1), *G. hirsutum* (HAU, version 1) and *G. barbadense* (HAU, version 1) were downloaded from the CottonFGD database (Zhu et al., 2017). The GT47 protein sequences of *A. thaliana* and *Populus trichocarpa* were downloaded from PlantCAZyme (Ekstrom et al., 2014). The hidden Markov model (HMM) proﬁle of the GT47 conserved domain (PF03016) was obtained from the Pfam database (Finn et al., 2016). The HMM file was used to query the GT47s in cotton, using the hmmsearch program of HMMER 3.0 software, with an E value threshold of 1.0 E-5 (Potter et al., 2018). Furthermore, the online Simple Modular Architecture Research Tool (SMART) was used to confirm the conserved domain for all the candidate GT47 protein sequences (Letunic et al., 2015).

### Chromosome Location and Collinearity Analysis

The chromosome location was displayed using MapChart software (Voorrips, 2002). For the collinearity analysis, the genome files of the four cotton species were used for sequence alignment with the Basic Local Alignment Search Tool (BLAST) with a cut-off E value of 1 × 10^−5^. MCScanX software was used to perform the collinearity analysis based on the results of BLASTP (Wang et al., 2012). The results were visualized using TBtools software, and the parameter for filtering genes in the small collinearity block was set to 40 (Chen et al., 2018).

### Evolutionary Selective Pressure

Homologous genes were determined based on the bidirectional BLAST results and the collinear chromosomal positions. The ratio of nonsynonymous (Ka) substitutions and synonymous (Ks) substitutions was used to assess the selection pressure of the homologous gene (Yang and Nielsen, 2000). The CDS sequence and protein sequence were used to ensure correct codon alignment. TBtools was used with NG methods to calculate the Ks/Ka ratios of homologous gene pairs (Chen et al., 2018).

### Sequence Alignment and Phylogenetic Analysis

ClustalX 2.0 (Thompson et al., 2002) was used for multiple sequence alignment of GT47 protein sequences with default parameters. The alignment result was used to construct an ML phylogenetic tree by PhyML 3.0 (Guindon et al., 2010). Bootstrap resamplings (100) were used to assess the reliability of interior branches. The substitution model (WAG model) and the rates among sites (Gamma distribution) were determined *via* automatic calculation with MEGA 7.0 software (Kumar et al., 2016). The online tool iTOL (https://itol.embl.de/) was used to display the phylogenetic tree (Letunic and Bork, 2016). To improve the reliability of the phylogenetic tree, SplitsTree4 (Huson and Bryant, 2006) was used to compute a phylogenetic network. The methods for character, distance and split parameters were set to Uncorrected_P, NeighborNet and EqualAngle, respectively. The bootstrap value was set to 1000.

### Physicochemical Features and Subcellular Location Prediction

The online Compute pI/Mw tool (http://www.expasy.ch/tools/pi_tool.html) was used to compute the isoelectric points (pI) and molecular weights (MWs) for each GT47 protein sequence. The N-terminal signal peptide and transmembrane helices were predicted by the SignalP 4.1 server and the TMHMM v.2.0 server, respectively (Krogh et al., 2001; Nielsen, 2017). Subcellular localizations of GT47 proteins were predicted by DeepLoc (Almagro Armenteros et al., 2017).

### Conserved Sequence and Gene Structure Analysis

The sequence logo of the exostosin (pf03016) domains was acquired using the online tool WebLogo (Crooks et al., 2004). The exon-intron structure analysis was performed with the online Gene Structure Display Server (GSDS) (Hu et al., 2015) by inputting gene annotation GFF files. The online MEME Suite was used to confirm the motifs of cotton GT47 protein sequences with the following parameters: a maximum number of six motifs and an optimum width of 6 to 50 (Bailey et al., 2006).

### Promoter Region Cis-Acting Element Analysis

The 2,000-bp upstream region of the initiation codon “ATG” of GT47s in *Gossypium spp* was used to perform the cis-acting element analysis with the online tool PlantCARE (http://bioinformatics.psb.ugent.be/webtools/plantcare/html/search_CARE.html) (Lescot et al., 2002).

### Plant Materials and Treatments

Three G. *hirsutum* cultivars (CG3020-3, Chuan338, and TM-1) were field grown in Anyang, Henan Province, China. CG3020-3 has long fibers (34.59 mm) and high fiber strength (34.3 cN/tex), while Chuan338 has short fibers (26.82 mm) and low strength (26.83 cN/tex). The fiber quality of approximately 10–15 g fiber samples was measured at the Cotton Fiber Quality Supervision, Inspection and Testing Center of the Ministry of Agriculture, Anyang, China. The roots, stems (near the shoot apical meristem), leaves (four-leaf-period cotton plants) and petals of TM-1 were harvested for RNA extraction. The fibers of CG3020-3 and Chuan338 were separated from the ovules 5, 10, 15, 20 and 25 days postanthesis (DPA) for RNA extraction. TM-1 was used for cold damage treatment of seedlings in a climate-controlled greenhouse (light/dark cycle: 16 h at 28°C/8 h at 22°C). Seedlings with two cotyledons were moved to an illumination incubator at 4°C. The whole plant was collected at 0 h, 1 h, 2 h, 4 h, 6 h, 8 h, 10 h and 12 h in the 4°C treatment. All samples were immediately frozen in liquid nitrogen and stored at −80°C.

### Transcriptome Data Analysis and Quantitative Real-Time Polymerase Chain Reaction (Qrt-PCR)

Raw RNA-seq data were downloaded from the NCBI Sequence Read Archive (SRA: PRJNA248163). TopHat2 (Kim et al., 2013) and cufflinks (Trapnell et al., 2010) were used to analyze RNA-seq expression, and gene expression was measured in fragments per kilobase million (FPKM). The TBtools program (Chen et al., 2018) was used to display the heatmap of gene expression. Total RNA was extracted from the collected samples using an RNAprep Pure Plant Kit (TIANGEN, Beijing, China) according to the manufacturer’s instructions. The first-strand cDNA was synthesized using a PrimeScript RT reagent kit (Takara, Dalian, China). Oligo 7.0 software was used to design the gene-specific primers for qRT-PCR (**Table S1**). Cotton histine 3 (GenBank accession no. AF094716) was used as an internal control (Tu et al., 2007). The qRT-PCR (Promega, Madison, WI, USA) on an ABI 7500 real-time PCR system (Applied Biosystems, USA) with three replicates. The 2-ΔΔCT method was used to calculate the relative expression levels of GhGT47s, and T-tests were used for statistical analysis.

## Results

### Identification and Chromosomal Distribution of GT47s in *Gossypium Spp.*

**Figure 1:**
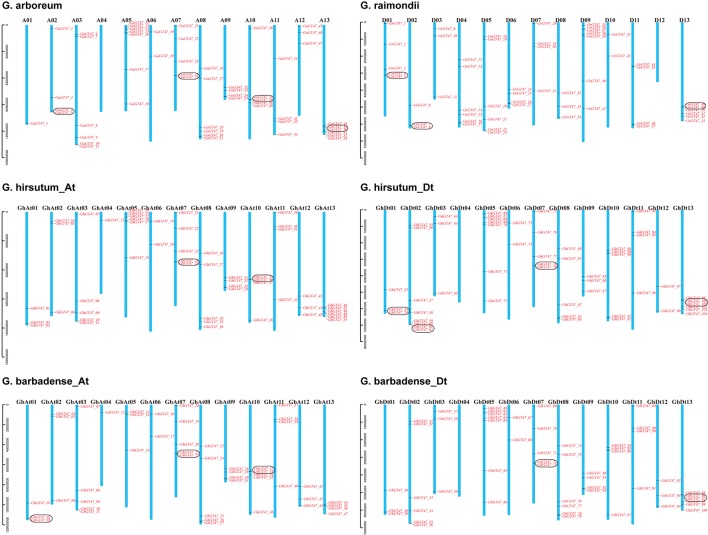
Chromosomal distribution of GT47s in *G. arboreum*, *G. raimondii*, *G. hirsutum* and *G. barbadense*. The chromosome numbers are indicated above each vertical bar. Tandem duplicated genes are marked by black outlined boxes.

A total of 53, 53, 105 and 109 putative GT47 sequences were detected by genome-wide identification analysis in *G. arboreum*, *G. raimondii*, *G. hirsutum* and *G. barbadense*, respectively. In *G. hirsutum* and *G. barbadense*, 1 and 9 GT47s were located on the scaffold fragments. The names of GT47s were determined according to their locations on the chromosomes (**Figure 1**), and the GT47s located on the scaffold fragments were ranked last in order. The location information is listed in **Table S2**. The chromosome location of each gene and the number of target genes for each chromosome are shown in **Figure 1**. In addition, tandem duplication gene pairs were determined by collinear analysis and marked by black boxes. In *G. arboreum*, *G. raimondii*, *G. hirsutum* and *G. barbadense*, there were 4, 3, 6 and 5 tandem duplication gene pairs, respectively. According to the comparison of the GT47 gene distribution in different cotton species, the GT47 gene family had a conserved chromosomal distribution as well as conserved gene numbers in *Gossypium*.

### Phylogenetic Analysis of GT47 Sequences

**Figure 2:**
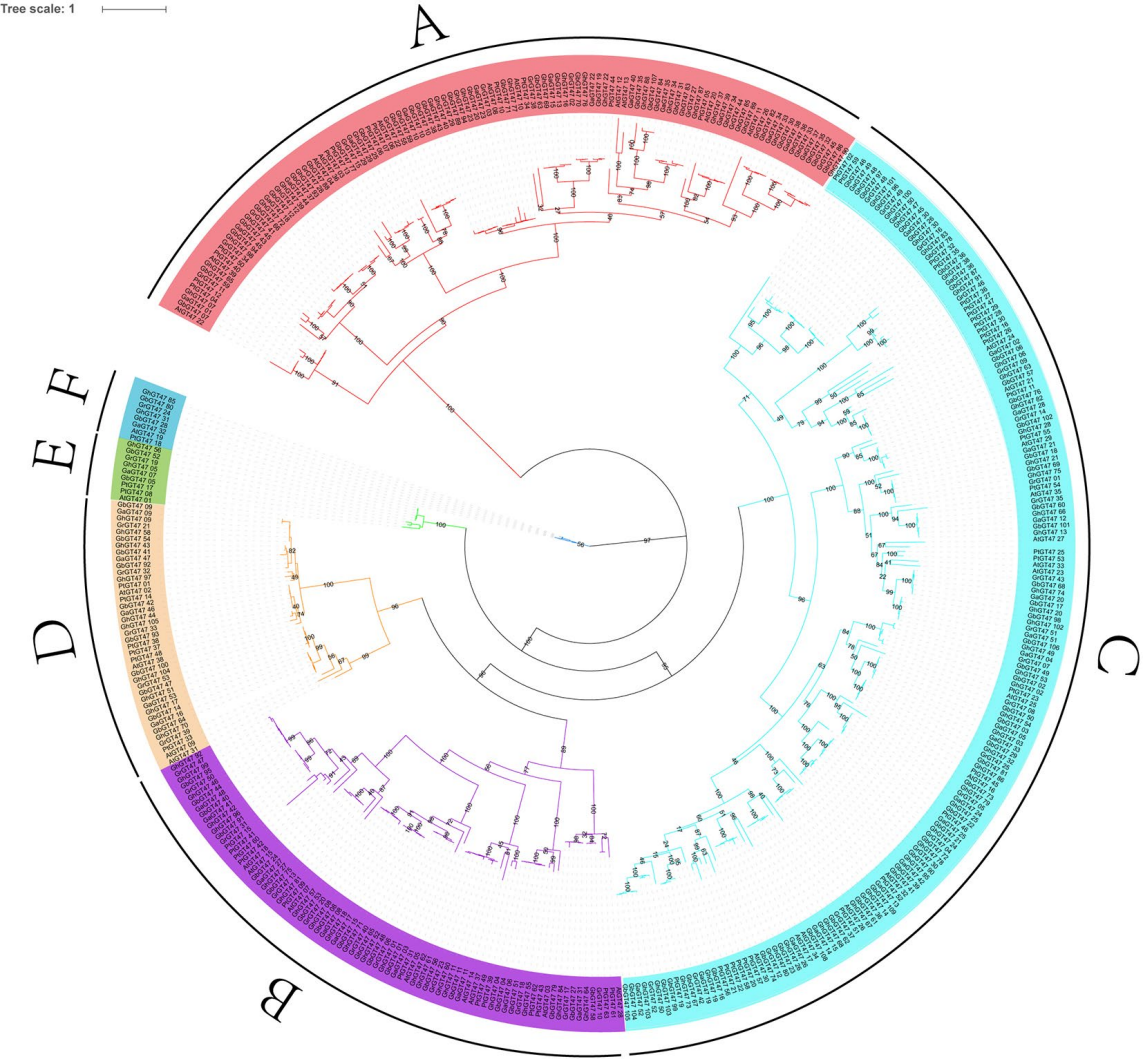
Phylogenetic tree of GT47 proteins. The 422 GT47 protein sequences of *G. raimondii*, *G. arboreum*, *G. hirsutum*, *G. barbadense*, *A. thaliana* and *P. trichocarpa* were aligned with ClustalX 2.0, and the phylogenetic tree was generated by PhyML 3.0 software using the ML method with 100 bootstrap replicates. Six subfamilies of GT47s are indicated using different line colors. The percentages of bootstrap numbers for the nodes are displayed on the branches.

To study the phylogenetic relationships of GT47s, a total of 422 GT47 protein sequences were used to construct an ML phylogenetic tree. These 422 GT47s were obtained from four cotton species, namely, *G. arboreum* (53), *G. raimondii* (53), *G. hirsutum* (105) and *G. barbadense* (109), and two other well-studied species, *A. thaliana* (39) and *P. trichocarpa* (63). The phylogenetic tree classified the GT47 family into six major groups, A-F (**Figure 2**), and the name of each subgroup was assigned according to previous results in *Arabidopsis* (Li et al., 2004, 3). The branches of the phylogenetic tree were consistent with the results of the phylogenetic network (**Figure S1**).

As shown in **Figure 2**, group C was the largest subgroup, containing 172 GT47s. In contrast, group E (9) and group F (8) had the smallest numbers of GT47 genes. In *G. hirsutum*, 105 GhGT47s were also divided into six groups, with 28, 21, 42, 10, 2 and 2 members in each subgroup.

### Evolution of GT47 Genes From Diploids to Allotetraploids

**Figure 3:**
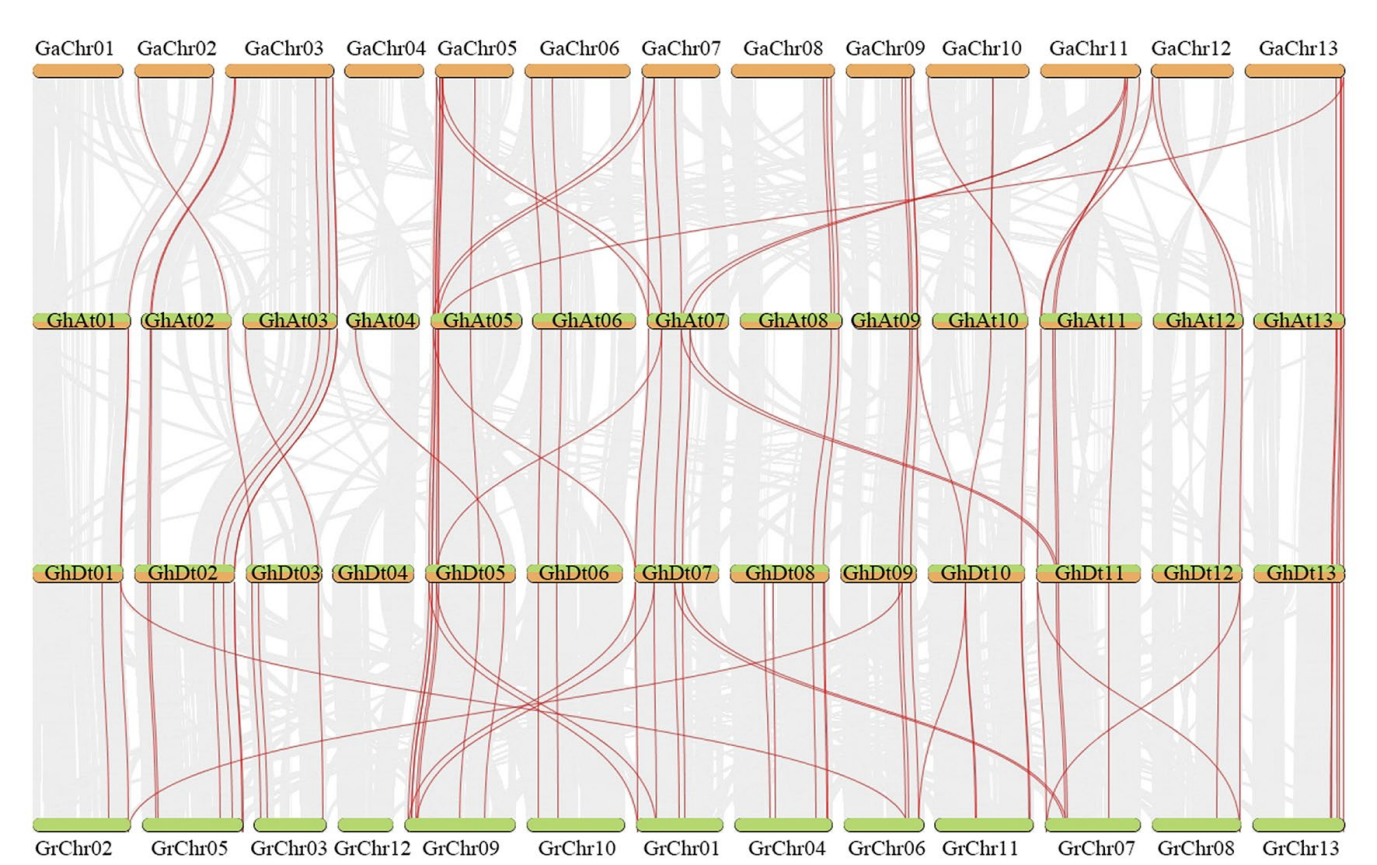
Collinear analysis between *G. arboreum* (AA), *G. hirsutum* (A and D subgenomes) and *G. raimondii* (DD) by MCScanX. Red and gray lines depict GT47 homologous gene pairs and WGD pairs, respectively.

The number of GT47s in allotetraploids (*G. hirsutum* (105) and *G. barbadense* (109)) is approximately equal to the sum of GT47s in *G. arboreum* (53) and *G. raimondii* (53). According to the evolution of cotton, tetraploid cotton is the result of two diploid cottons that hybridized, followed by chromosome doubling. We infer that the GT47 family evolved from diploid cotton to tetraploid cotton mainly through whole-genome duplication (WGD). To confirm this inference, collinearity analysis was performed using MCScanX between the At and D subgenomes of *G. hirsutum* and their corresponding ancestral A and D diploid genomes (**Figure 3**). From the collinearity results, we found that most GT47 homologous gene pairs between different genomes were contained in the collinear blocks. The same characteristics were found in *G. barbadense* (**Figure S2**). This result provides reliable evidence for the inference that the GT47 gene family expanded in *Gossypium* mainly *via* WGD or segmental duplication. The duplicate types of GT47s in *G. hirsutum* and *G. barbadense* were identified by MCScanX and are shown in **Table S3**.

The genome of allotetraploid cotton species is very complex. During the process of evolution, the genome experienced not only whole-genome replication events but also many repeated events, resulting in the presence of many duplicative gene pairs in the cotton genome. In this study, we mainly concentrated on the homologous genes between species and subgenomes. The homologous gene pairs were identified by the results of BLAST and collinearity analysis (**Figure 3** and **Table S4**). As shown in **Table S4**, a total of 79 homologous gene pairs were identified between *G. arboreum* and the corresponding A subgenomes of *G. hirsutum* and *G. barbadense*, containing 49 GaGT47s (**Table S4**). There were 16 speciﬁc gene pairs between *G. arboreum* and the *G. hirsutum* A subgenome and only three specific gene pairs between *G. arboreum* and the *G. barbadense* A subgenome. The Ka/Ks ratio was calculated to assess the selection pressure of these homologous gene pairs. Between these 79 homologous gene pairs, 64 gene pairs had Ka/Ks <1, indicating that most homologous gene pairs experienced purifying selection during the process of evolution. Between the D subgenomes, there were 93 gene pairs containing 50 GrGT47s. Three GrGT47s had homologous gene pairs only in *G. hirsutum*, and four GrGT47s had homologous gene pairs only in *G. barbadense*. Most of these (84/93) gene pairs experienced purifying selection (Ka << Ks). The comprehensive results showed that most GT47s experiencing evolution, due to the effects of genome-wide replication, will be strongly purified by selection pressure, and these genes may retain their original functions. Only a few GT47s undergo positive selection, which may lead to the production of new biological functions of these genes.

### Physicochemical Features and Subcellular Location Prediction of GT47s

The calculated pI and MW of each GT47 are shown in **Table S5**. The average molecular weight of *G. arboreum*, *G. raimondii*, *G. hirsutum*, *G. barbadense*, *A. thaliana* and *P. trichocarpa* was 59,086.83 Da, 59,178.90 Da, 57,203.30 Da, 57,487.23 Da, 58,602.37 Da and 53,637.21 Da, respectively. The statistical results showed that the MWs of diploid ancestor cottons were larger than those of allopolyploid cotton species. The SignalP 4.1 server was used to identify signal peptides (Nielsen, 2017). A total of 39 GT47s containing signal peptides were detected in *G. arboreum* (5), *G. raimondii* (6), *G. hirsutum* (14), *G. barbadense* (14), *A. thaliana* (3) and *P. trichocarpa* (5). Most GhGT47s (82/105) harbored a putative transmembrane domain, among which 74 GhGT47s harbored one transmembrane domain, six GhGT47s harbored two transmembrane domains, and two specific genes, *GhGT47_19 and GhGT47_50*, contained six and seven transmembrane domains, respectively. The subcellular localization results showed that most of the GhGT47s were targeted to the Golgi apparatus (64) and endoplasmic reticulum (24), as predicted by DeepLoc, suggesting that the Golgi apparatus was the main area where GT47s had biological functions.

### Conserved Sequence and Gene Structure Analysis

**Figure 4:**
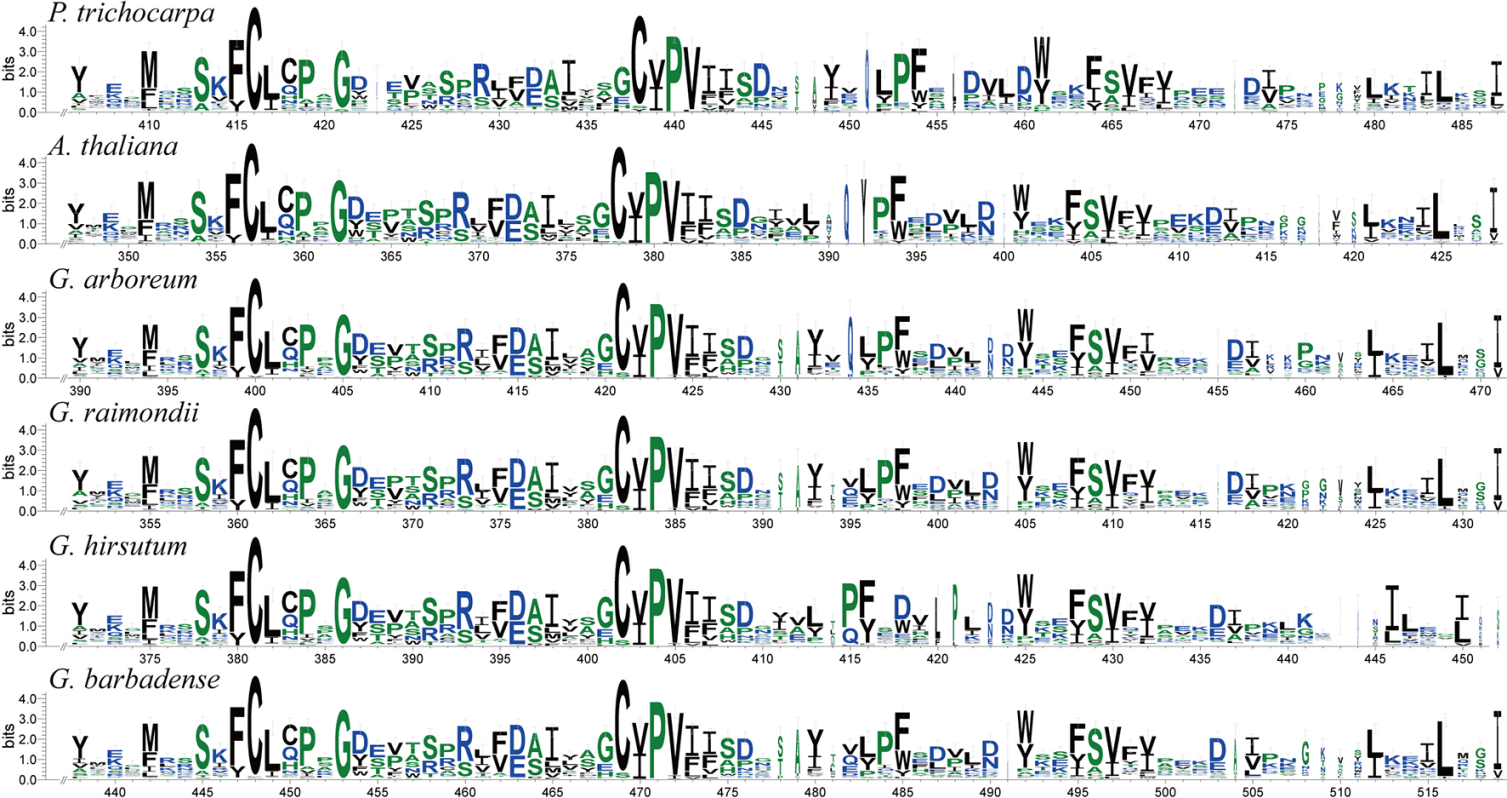
Sequence logos of the partial conserved domain (exostosin) in different species. The sequence logos were determined by the WebLogo program.

To analyze the homologous domain sequences and degree of conservation of exostosin domains, exostosin domain sequences were obtained from all the predicted GT47s and used to construct sequence logos. The results revealed multiple conserved fragments in the full-length logos of exostosin domains (**Figure S3**). Partial sequence logos are shown in **Figure 4**, indicating that the exostosin domains were signiﬁcantly conserved in GT47s among multiple species.

**Figure 5:**
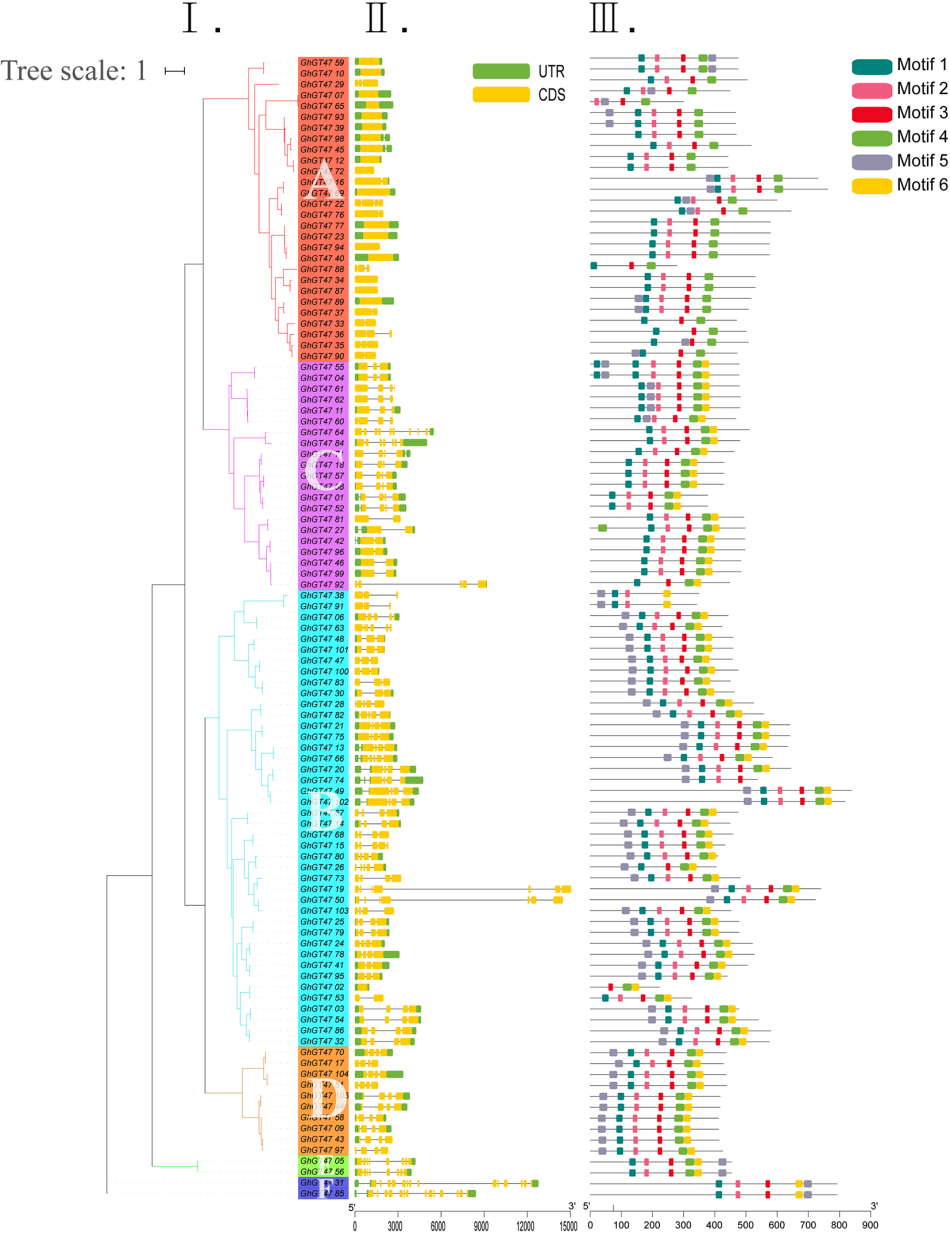
Gene structure and conserved protein motifs of GhGT47s. I. ML phylogenetic tree analysis of *G. hirsutum*. A-F represent the six subgroups. II. The number, length, and position of exons and introns within GhGT47 genes. Boxes indicate exons, and green lines indicate introns. III. Distribution of the predicted motifs in the GhGT47 genes.

To better understand the structural features of GhGT47s, their gene exon/intron structures were analyzed using the online GSDS program. The phylogenetic analysis results showed that 105 GhGT47s were divided into six groups (A-F) containing 28, 21, 42, 10, 2, and 2 members (**Figure 5I**). The exon/intron structures of GhGT47s are shown in **Figure 5II**. The GhGT47 genes contained multiple numbers of exons ranging from 1 to 14, but most contained fewer than five exons. The exon/intron structure of group A was more conserved than that of the other groups. Further analysis of the conserved motif was performed using the MEME program. Six conserved motifs were predicted, and sequence logos of six motifs were obtained (**Figure 5III**; **Figure S4**). As shown in **Figure 5III**, GhGT47s in the same group displayed a similar motif composition, which further supported the group classification result. Motif 1, motif 2, motif 3, motif 4, and motif 6 were arranged in the same order within most GhGT47s, while motif 5 appeared first or last in the sequence. **Figure 5** shows that the exon/intron structure and motif distribution differed among different groups, while on the same branches, they were highly conserved. We also performed exon/intron structure and motif analysis of all 422 GT47s (**Figure S5**). The results showed obvious conservation, laying a foundation for functional conservatism and providing guidance for subsequent functional research.

### Analysis of Cis-Acting Elements in GhGt47 Gene Promoter Regions

**Figure 6:**
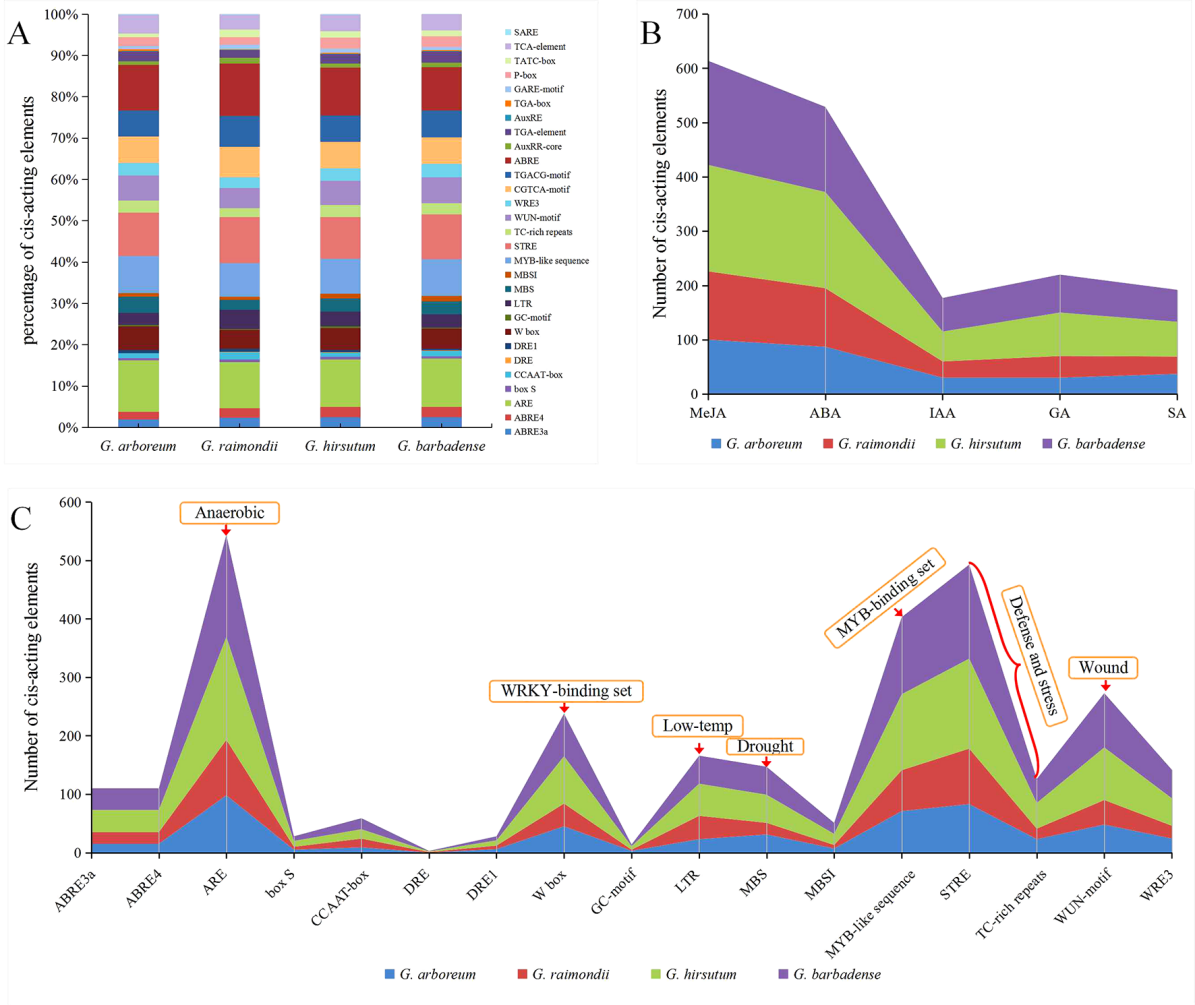
Distribution characteristics of stress-related and phytohormone-responsive cis-acting elements in GT47 gene promoter fragments. The cis-acting elements were identified by PlantCARE using the upstream 2000-bp sequences of the GT47 genes. **(A)** The proportion of each cis element in four different cotton species. **(B)** The sum of cis-acting elements related to methyl jasmonate (MeJA), abscisic acid (ABA), auxin (IAA), gibberellin (GA) and salicylic acid (SA) in the GT47 promoter fragment of four cotton species. **(C)** The specific number of stress-related cis-acting elements in each of four cotton species, and the functions of some significant cis-acting elements are indicated.

The analysis of cis-acting elements of promoters is an important research method to understand the regulation of gene transcription and expression. To explore whether GT47s in different species have the same regulatory mechanism, the upstream 2000-bp sequences of the initiation codons of cotton 320 GT47s were used for cis-acting element identification by PlantCARE (Lescot et al., 2002). These stress-related and phytohormones-responsive cis-acting elements were our focus (**Table S6**, **Figure 6**). As shown in **Figure 6A**, different cis-acting elements of the GT47 gene promoter fragment had the same proportions in different cotton species, indicating a similar regulatory mode of GT47 genes in different cotton species.

The statistical results for the cis-acting elements indicated that the regulation of GT47 genes was mainly related to the following phytohormones, including methyl jasmonate (MeJA), abscisic acid (ABA), auxin (IAA), gibberellin (GA) and salicylic acid (SA). **Figure 6B** shows the sum of cis-acting elements related to the same phytohormones in the GT47 promoter fragment of four cotton species, among which cis-acting elements that respond to MeJA and ABA hormones were the most common.

In addition, these stress-related cis-acting elements were also a focus of attention. As shown in **Figure 6C**, these stress-related cis-acting elements were mainly related to anaerobic, defense, drought, low temperature, wound and other stresses, among which anaerobic-related (ARE) cis-acting elements were the most common, followed by those defense-related elements (STRE and TC-rich repeats) and the binding set of the MYB transcription factor (MYB-like sequence). The results indicate that the genes in the GT47 family may be related to various abiotic stresses. It follows that GT47s may increase the resistance of cotton. The cis-acting element results suggest that GT47s may be regulated by multiple phytohormones and related to multiple stresses in *Gossypium spp.*

### Expression Characterization of GhGt47s in Different Tissues

**Figure 7:**
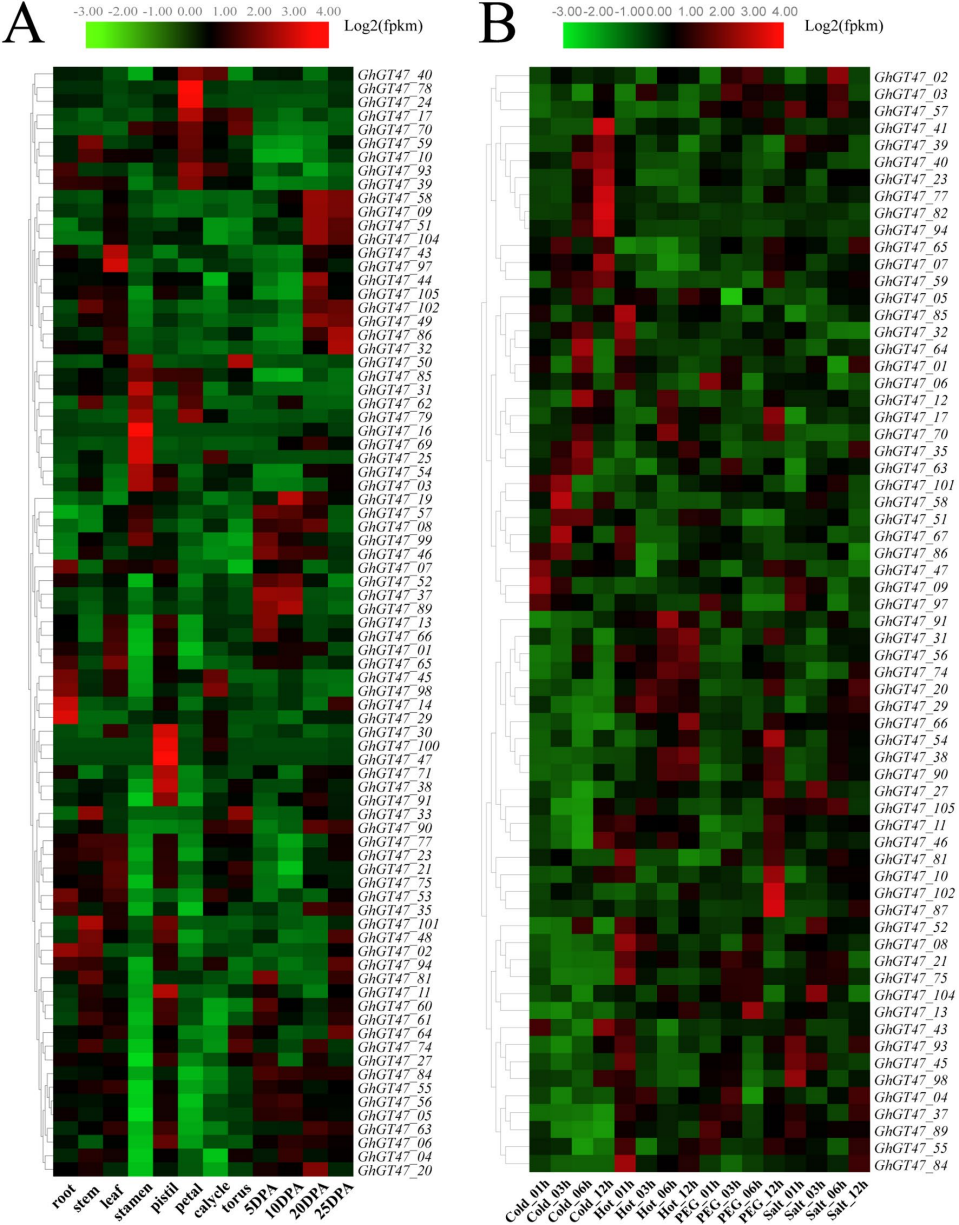
Expression profiles of GhGT47s in different tissues **(A)** and in response to different stresses **(B)**. The tissues or treatments are shown at the bottom, genes are shown on the right, and the phylogenetic relationships are shown on the left.

Expression pattern analysis is helpful to predict the biological functions of genes, and the spatiotemporal expression pattern of GhGT47s was investigated based on transcriptome data of multiple tissues, including the root, stem, leaf, stamen, pistil, petal, calyx, torus and four fiber developmental stages. As shown in**Figure 7A**, GhGT47s were widely expressed in different tissues, and most GhGT47s were expressed in a single tissue, suggesting that GhGT47s has multiple biological functions that show tissue specificity. GhGT47s were mainly expressed in the reproductive organs, such as the stamen, pistil and petal. Only a few genes were expressed preferentially in the vegetative organs. *GhGT47_02*, *GhGT47_14, GhGT47_29* and *GhGT47_98* were expressed at relatively high levels in the root, and *GhGT47_101* was highly expressed in the stem. Only a pair of genes (*GhGT47_97*/*GhGT47_43*) were highly expressed in leaves.

**Figure 8:**
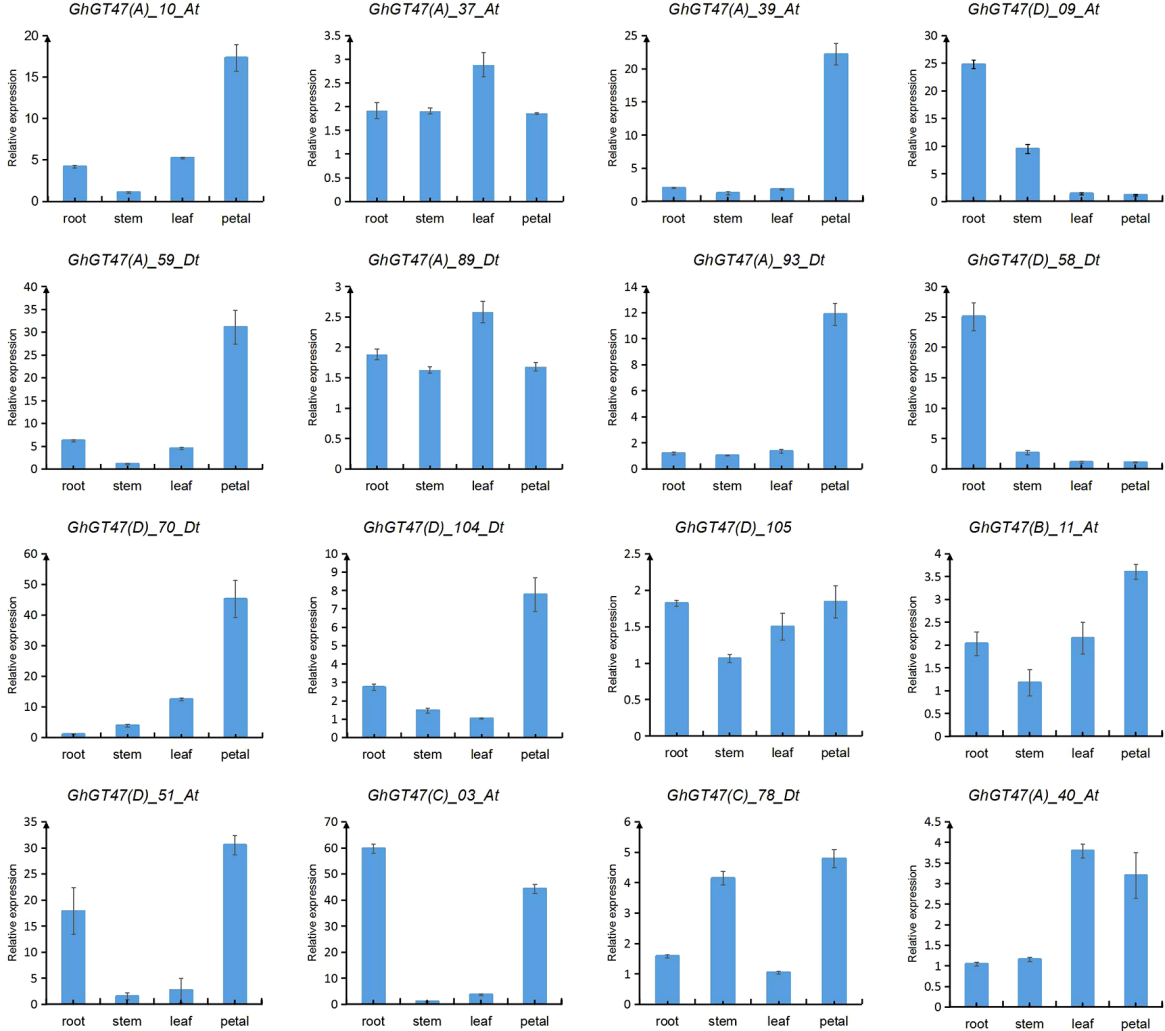
Expression analysis of GhGT47s in different tissues. Error bars show the standard deviation of three biological replicates. The letters in parentheses after the gene name represent the subgroup to which it belongs.

Based on the transcriptome data, several genes of interest were selected for further verification by qRT-PCR. The gene pairs (*GhGT47_10*/*GhGT47_59*, *GhGT47_37*/*GhGT47_89*, *GhGT47_39*/*GhGT47_93* and *GhGT47_09*/*GhGT47_58*) were homologous gene pairs located on At and Dt. These gene pairs exhibited the same expression pattern (**Figure 8**). Most genes were highly expressed in the petal, except for *GhGT47_09*/*GhGT47_58*. The qRT-PCR results showed that the *GhGT47_09*/*GhGT47_58* genes were highly expressed in the root. In summary, the qRT-PCR results show that GhGT47s are widely expressed in different tissues and have different expression patterns. This phenomenon further explains the functional diversity and expression-site specificity of GT47 genes.

### Expression Characterization of GhGt47s in Fiber Development

To further analyze the correlation between GhGT47 genes and fiber development, the expression pattern of fiber development in different stages was examined. As shown in **Figure 7A**, most of the GhGT47s were highly expressed at 10 DPA and 20 DPA, and the GhGT47s that were highly expressed at 10 DPA generally belonged to group A, while those that were highly expressed at 20 DPA generally belonged to group D. This phenomenon implied that GhGT47 genes in the same group might have similar functions. Previous studies have shown that several genes in subgroup A are involved in xyloglucan synthesis, and subgroup D genes are involved in xylan synthesis (Tamura et al., 2005, 3; Brown et al., 2009, 10). According to the expression characteristics of GT47 at different stages of fiber development, we can infer that the corresponding polysaccharide molecules may be needed at different stages of fiber development.

**Figure 9:**
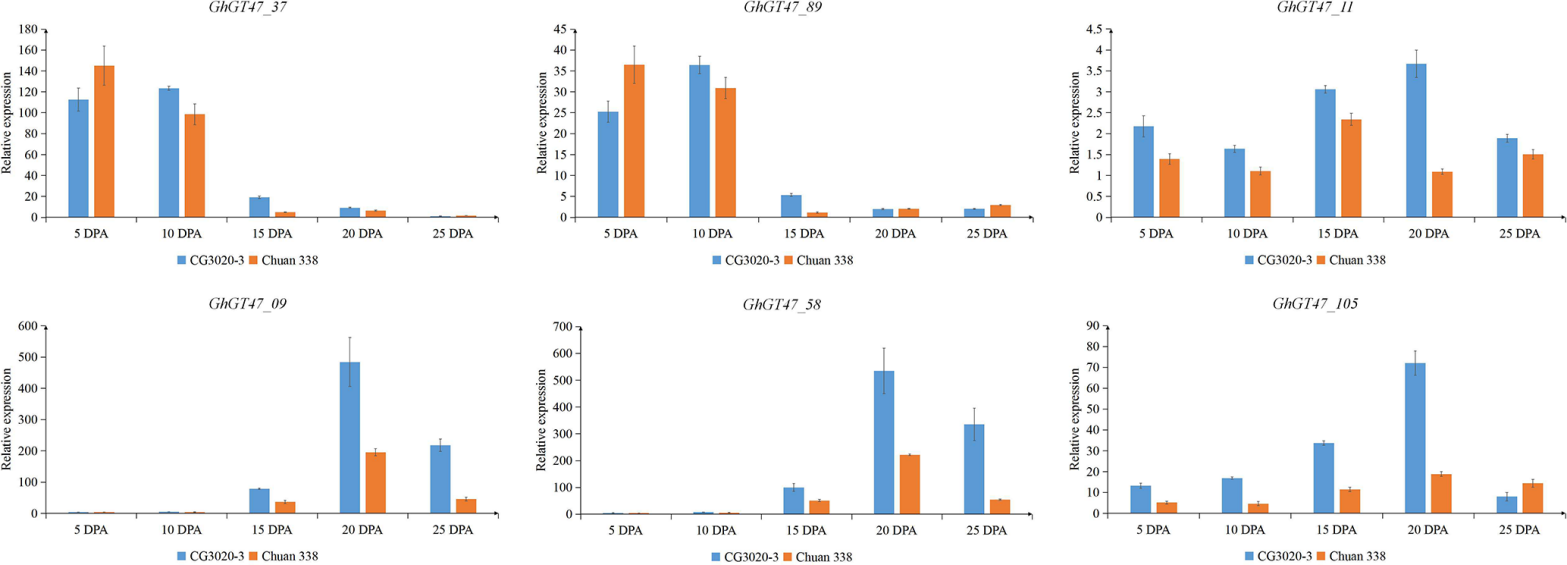
Relative expression of GhGT47s at different fiber development stages. Error bars show the standard deviation of three biological replicates. CG3020-3 (longer fibers) and Chuan338 (shorter fibers) are two cultivars with significantly different fiber lengths. DPA (days postanthesis) is the sampling period.

To explore whether GhGT47s are related to fiber length, two cultivars with significantly different fiber qualities, namely, CG3020-3 (longer fibers and a greater strength) and Chuan338 (shorter fibers and lower strength), were chosen for further analysis. *GhGT47_37*, *GhGT47_89*, *GhGT47_09*, *GhGT47_58*, *GhGT47_105* and *GhGT47_11* were selected to perform qRT-PCR in the two cultivars. As shown in **Figure 9**, *GhGT47_37* and *GhGT47_89* had high expression levels during early elongation of the fibers (5-10 DPA) that decreased rapidly after 15 DPA. The relative expression levels of *GhGT47_37* and *GhGT47_89* were significantly different between the two cultivars, suggesting that *GhGT47_37* and *GhGT47_89* were related to fiber length. The expression level of *GhGT47_11* was higher in CG3020-3 than in Chuan338 at all stages. The expression of *GhGT47_11* was highest in CG3020-3 20 DPA and in Chuan338 at 15 DPA. *GhGT47_09* and *GhGT47_58* belonged to the D subgroup. As shown in **Figure 9**, *GhGT47_09*, *GhGT47_58* and *GhGT47_105* were highly expressed at 20 DPA and 25 DPA, and the expression was higher in CG3020-3 than in Chuan338. In summary, the expression of these six genes was positively correlated with fiber length and strength, providing candidate genes for studies aiming to improve fiber quality.

### Expression Characterization of GhGt47s Under Multiple Stress Treatments

The results of the promoter region cis-acting element analysis showed that GhGT47s might be related to stress response, and some studies have suggested that the GT47 genes have a positive effect on resistance (Bethke et al., 2016; Tan et al., 2018, 47). Therefore, we further analyzed the GhGT47 expression patterns under multiple stresses. As shown in **Figure 7B**, several genes had relatively high expression levels under different stresses. Most GhGT47 genes responded to cold stress, especially in the 12 h cold treatment. We found that different genes started responding at different treatment times, for example, *GhGT47_58* and *GhGT47_67* were expressed at relatively high levels in the 3 h cold treatment, while the expression level began to decline when the experimental treatment time was extended. Simultaneously, other GhGT47s began to exhibit increased expression (*GhGT47_41*, *GhGT47_77*, *GhGT47_82* and *GhGT47_94*). In addition to responding to the cold treatment, several GhGT47s responded to polyethylene glycol (PEG) treatment and salt treatment, suggesting that GhGT47s could improve the resistance of plants to abiotic stresses and provide potential candidate genes for further study.

**Figure 10:**
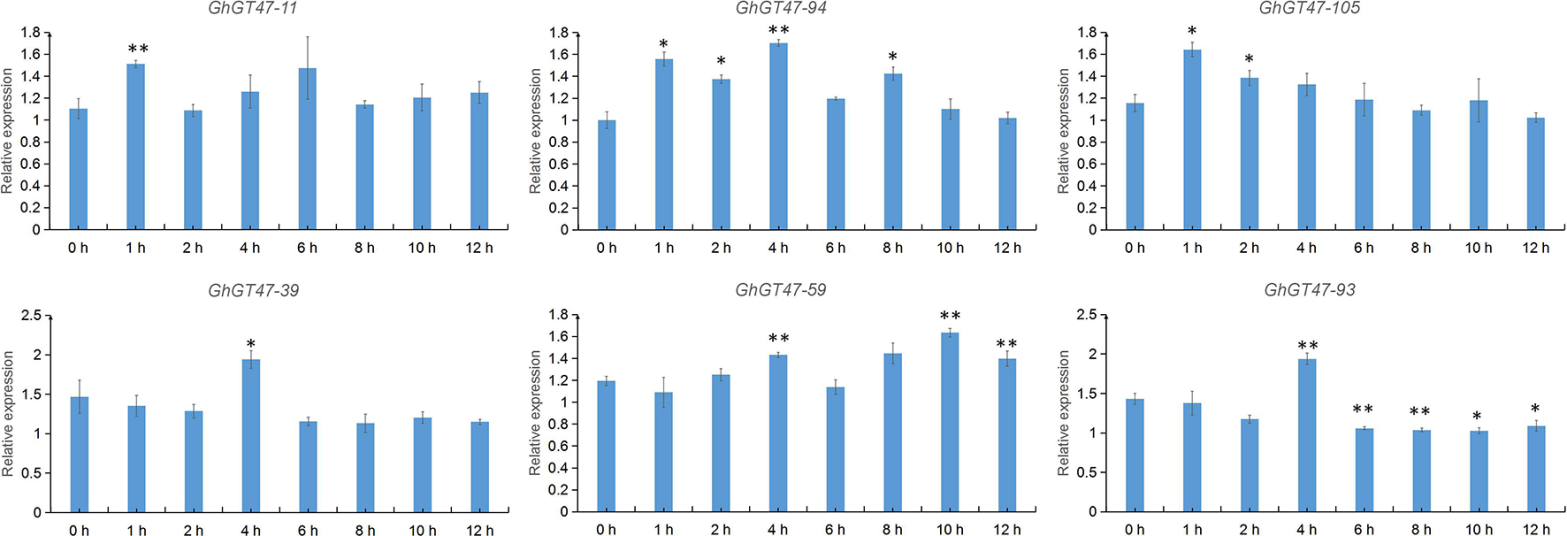
Expression analysis of GhGT47s under cold treatment *via* qRT-PCR. The 1 h, 2 h, 4 h, 6 h, 8 h, 10 h and 12 h indicate h after cold treatment, with 0 h representing the control sample. Single and double asterisks represent significant differences from the control sample at the 0.05 and 0.01 levels, respectively. Error bars show the standard deviation of three biological replicates.

Based on previous studies and transcriptome data, *GhGT47_11*, *GhGT47_94*, *GhGT47_105*, *GhGT47_39*, *GhGT47_59* and *GhGT47_93* were selected to perform qRT-PCR. As shown in **Figure 10**, cold stress regulated the activity of GhGT47s. *GhGT47_11*, *GhGT47_94* and *GhGT47_105* responded to cold treatment at 1 h, and *GhGT47_39*, *GhGT47_59* and *GhGT47_93* began to respond at 4 h. The expression of *GhGT47_11*, *GhGT47_94* and *GhGT47_105* increased after 1 h of treatment; *GhGT47_94* maintained a high expression level at 2 h, 4 h and 8 h; and *GhGT47_11* and *GhGT47_105* showed no significant difference from the control group. *GhGT47_59* was the only gene with high-level expression at 10 h and 12 h of cold treatment. *GhGT47_93* exhibited high-level expression at 4 h; however, after 4 h, the expression began to decrease.

## Discussion

GTs participate in the anabolic metabolism of carbohydrates in living organisms by transporting an active sugar molecule to a specific acceptor. The function of GTs provides the basic guarantee of maintaining normal physiological activities. These molecules exist widely in living organisms, especially plants. Multiple GT genes work together to synthesize complex polysaccharide molecules. In plants, GTs are responsible for the synthesis of polysaccharide chains of the cell wall and complex sugars (glycoproteins, glycolipids), and they are involved in the cellular framework and the regulation of physiological processes (Keegstra and Raikhel, 2001). Multiple GT families have been identified and verified in different plants (Vuttipongchaikij et al., 2012; Zhou et al., 2006; Huang et al., 2008; Zeng et al., 2010; Mamoon Rehman et al., 2016). However, few studies have been performed to examine GT genes in cotton, especially the GT47 family. The GT47 gene family belongs to the GTs and is grouped based on the β-glucuronyltransferase domain (Zhong and Ye, 2003). In the current study, we performed a comprehensive bioinformatics analysis of the GT47 genes in cotton. Specifically, GT47 family members were identified through sequence similarity and then analyzed for the evolutionary amplification mode, sequence features, and expression mode, among others. More comprehensive analyses of the characteristics of the GT47 gene family in cotton will provide a basis for further studies.

### Gene Expansion of GT47s in Cotton

Polyploidy is pervasive in plants and has previously been identiﬁed in more than 90% of ﬂowering plants. During the process of evolution, polyploidization is a major mechanism of adaptation and speciation in plants (Ramsey and Schemske, 1998). The duplication of single genes, chromosomes and whole genomes is a major force in the evolution of plant genome structure and content (Paterson et al., 2012). The allotetraploid species *G. hirsutum* L. has experienced many duplication events, and the gene number has enlarged during evolution, making it an ideal plant for studying the role of polyploid formation in evolution (Paterson et al., 2012; Zhu and Li, 2013). Diploid cotton with the A-genome is native to Africa, and D-genome diploid cotton is native to Mexico. These two diploid ancestral species, each containing A and D subgenomes, hybridized *via* transoceanic dispersal approximately 1-2 MYA followed by chromosome doubling, from which the nascent allopolyploid *Gossypium* species was formed (Wendel and Cronn, 2003; Li et al., 2015). In this study, a total of 53, 53, 105 and 109 putative GT47 sequences were identified by genome-wide analysis in *G. arboreum*, *G. raimondii*, *G. hirsutum* and *G. barbadense*, respectively. A comparison of the *G. hirsutum* genome with the *G. arboreum* (AA) and *G. raimondii* (DD) genomes revealed that the chromosomal distribution and gene numbers were conserved in GT47s between diploid ancestral species and tetraploid cotton species. The number of GT47 genes in tetraploid cotton species is approximately equal to the sum of *G. arboreum* (53) and *G. raimondii* (53). The collinearity analysis showed that most GT47 homologous gene pairs between the A and D subgenomes of *G. hirsutum* and their corresponding A and D diploid genomes were located in collinear blocks. These results indicated that WGD was the major driver of the expansion of GT47 genes from diploids to allotetraploids.

### Evolutionary Conservation and Divergence of GT47 Genes

The conservation of structural sequences is the basis of biological functional conservation. The GT47 family was identified based on the β-glucuronyltransferase domain (Zhong and Ye, 2003). To verify the conservation of the GT47 gene sequences, we performed exon/intron structure analysis and motif prediction (**Figure 5**, **Figure S5**). The results showed that the gene structures and motifs of these GT47s in the same branch had similar characteristics. In addition, all the sequences of conserved domains were used to perform a sequence logo analysis. As shown in this study (**Figure 4**, **Figure S3**), the full-length logo of the exostosin (pf03016) domains was characterized by several highly conserved segments, and the relative positions of each conserved segment were similar in different species. The conserved sequences of GT47s were highly conserved in different species. Sequence conservation is the basis of functional conservation. Thus, the functions of cotton GT47s can be inferred according to the biological functions of corresponding homologous genes in other species. The functions of homologous genes provide a basis and direction for later functional research.

During the process of evolution, divergence must occur. The ratio of nonsynonymous (Ka) substitution to synonymous (Ks) substitution was used to assess the selection pressure of the homologous genes. The Ka/Ks ratios of these homologous were calculated between the At and D subgenomes of allotetraploid cottons and their corresponding A and D diploid genomes (**Table S4**). Most Ka/Ks values of GT47 homologs were less than 1, and we speculated that the GT47 gene family was subjected to purifying selection during the long-term evolutionary process, resulting in segmental gene duplication and WGD, which could maintain the original function of the gene.

### Cis-Acting Elements of GT47s in Upland Cotton

The expression regulation of eukaryotes is multilevel, and transcriptional regulation is the main mechanism of plant gene expression regulation; the interaction between promoter binding sites and transcription factors plays an important role in regulation at the transcriptional level. Inducible promoters are induced and activated by external signals, and cis-acting elements of inducible signals in promoters have specificity and consistency. For example, light-induced promoters generally contain G-box, I-box, GT1-motif and AT-rich cis-acting elements (Lam and Chua, 1989; Gilmartin et al., 1992; Foster et al., 1994), and auxin-induced promoters generally contain cis-acting elements of AuxRE, DR5, etc. (Ulmasov et al., 1997), while drought-induced promoters generally contain CATGTG and CACG cis-acting elements (Tran et al., 2004). In the present study, upstream promoter fragments of candidate genes were extracted, and statistical analysis of the cis-acting elements was performed. Those cis-acting elements related to stresses and phytohormones were our focus, as shown in **Table S6** and **Figure 6**.

As shown in **Figure 6B**, these phytohormone-responsive cis-acting elements mainly respond to several phytohormones, including MeJA, ABA, IAA, GA and SA. A large number of studies have shown that various phytohormones are associated with maintaining the integrity of plant cell walls and even play important roles. MeJA plays a role in mediating gene expression associated with plant responses to injury (Creelman et al., 1992), and auxin is involved in the regulation of cell wall properties by inducing cell wall looseness (Majda and Robert, 2018). Ethylene can regulate the synthesis of pectin in strawberry cells and promote fruit softening and ripening (Villarreal et al., 2016). Brassinosteroid (BR) signaling is essential to ensure the homeostasis of cell wall biosynthesis and remodeling (Wolf et al., 2012). According to the statistical results and existing experimental conclusions, the GT47 gene may be regulated by various phytohormones.

Moreover, a total of 2793 stress-related cis-acting elements were identified and divided into several categories (**Table S6**, **Figure 6C**), including defense and stress (STRE, TC-rich repeats), MYB binding region (MYB-like sequence), WRKY binding site (W box), heat (HSE), drought (MBS, DRE1), anoxia (GC-motif and ARE), low temperature (LTR and DRE) and wounding (WUN-motif, WRE3 and box S). In addition, the proportion of each cis-acting element in different cotton species was similar (**Figure 6A**), indicating that the genes in the GT47 family have the same regulatory mode in cotton, which also confirms the evolutionary conservation of the GT47 family in cotton. The cis-acting element results suggested that GhGT47 genes might be regulated by multiple hormones and related to multiple stresses, and they might be beneficial by enhancing resistance to stress in *Gossypium spp*. The cell wall is the first physical barrier for plants to resist the outside world. These stress-related cis-acting elements give plants the ability to respond quickly and regulate the expression of related genes, thereby improving their resistance to the outside world.

### The Function of GT47s in Upland Cotton

The GT47 gene family has been identiﬁed in multiple taxa, such as *Arabidopsis*, rice, and sorghum (Zhong and Ye, 2003; Cao et al., 2008; Xu et al., 2018, 47), and the functions of some family members have become clear. *MUR3* is a representative GT47 gene of group A, which encodes a xyloglucan galactosyltransferase that specifically catalyzes the XXXG core structure of xyloglucan to XXLG subunits (L side chain) by transporting the Gal residue to the third xylose residue in *Arabidopsis* (Madson et al., 2003, 3). Xyloglucan is the primary hemicellulose in the primary cell wall of dicotyledons (Pauly and Keegstra, 2016). Overexpression of the *MUR3* homologous genes *SbGT47_2* and *SbGT47_7* of sorghum in the *Arabidopsis mur3-3* mutant increases the XXFG and XLFG content (Xu et al., 2018, 3). In the *xlt2mur3.1* mutant of *Arabidopsis*, overexpression of the *MUR3* homologous genes of tomato or rice could recover the dwarfed growth phenotype of plants (Liu et al., 2015). These results suggest that the function of the *MUR3* gene is conserved in different species. In the present study, two homologous genes (*GhGT47_37*/*GhGT47_89*) of *ATMUR3* were identified in *G. hirsutum*. The qRT-PCR results showed that *GhGT47_37*/*GhGT47_89* had high expression during the early elongation of fibers (5-10 DPA), which is the main period of primary cell wall synthesis (**Figure 9**). The present results indicated that the gene expression trend was consistent with the expected gene function. Moreover, this period is the key period of fiber elongation. After comprehensive consideration of the gene sequence similarity, expression pattern and fiber development characteristics, we deduced that *GhGT47_37/GhGT47_89* might have a conserved function with *ATMUR3* and might be involved in the synthesis of xyloglucan during fiber elongation, providing raw materials for the primary cell wall to enhance fiber length. The detailed functions must be further verified in cotton.

*IRX10* and *IRX10L* were identified as xylan xylosyltransferases and involved in the synthesis of the xylan backbone chain together with *IRX9* and *IRX14* (Rennie and Scheller, 2014). These genes performed critical functions in the synthesis of glucuronoxylan during secondary cell wall formation (Brown et al., 2007; Wu et al., 2009). *OsGT47A* is the homologous gene of *AtIRX10*, exhibits a conserved function and is most likely involved in xylan synthesis in rice (Zhang et al., 2014). In *G. hirsutum*, we identified 4 *IRX10* homologous genes and 1 *IRX10L* homologous gene. Combined with transcriptome data and qRT-PCR, the results showed that two *Gh-IRX10s* (*GhGT47_09/GhGT47_58*) and one *Gh-IRX10L* (*GhGT47_105*) were highly expressed at 20 DPA and 25 DPA (**Figure 9**). During this period, the fiber cell wall began to change from the synthesis of primary cell wall to secondary cell wall, and hemicellulose began to deposit and thicken the cell walls (Haigler et al., 2012). *GhGT47_09/GhGT47_58* and *GhGT47_105* may be involved in the synthesis of secondary cell walls. Based on a comparison of the expression in two cultivars with significantly different fiber lengths and strengths, the expression levels of the three genes were higher in the high-quality fiber variety (**Figure 9**). Taken together, these results highlight the importance of *GhGT47_09/GhGT47_58* and *GhGT47_105* in the synthesis of secondary wall fibers. Through this thickening process, the secondary cell wall fiber strength is increased.

*GhGT47_11* is the homologous gene of *AtARAD1*, which is involved in the synthesis of pectin in the cell wall (Harholt et al., 2012). Pectin is the main material filling the intercellular space and plays an important role in cell wall elongation (Caffall and Mohnen, 2009). Additionally, it was expressed at higher levels in long-fiber cultivars than in short-fiber cultivars in all stages of fiber development (**Figure 9**). Furthermore, the period of highest expression was delayed compared with that in short-fiber varieties. This phenomenon indicated that *GhGT47_11* played an important role in fiber development.

In previous studies, GT47s have also been shown to be associated with abiotic stress and biotic stress (Bethke et al., 2016; Tan et al., 2018). Plant resistance to the outside environment is a complex process, including cell wall physical blockade, hormone responses, and ion changes, among others. In terms of the physical barrier of the cell wall, there are two main mechanisms: i) increasing the cell wall thickness by increasing the hemicellulose and lignin contents; ii) increasing xyloglucan endotransglucosylase/hydrolase (*XTH*) and expansin proteins (Le Gall et al., 2015). Previous studies have shown that the GT47 family is one of the main gene families involved in cell wall polysaccharide synthesis. GT47s are essential for maintaining normal cell wall biological functions and increasing resistance to environmental stress. In recent years, multiple studies on cold stress have shown that some of the cold-responsive genes are involved in cell wall metabolism based on comparative transcriptome analyses in *Prunus persica* (Nilo-Poyanco et al., 2019), bell pepper (Zuo et al., 2018) and tobacco (Hu et al., 2016). In cotton, a transcriptome analysis of common differentially expressed genes under multiple abiotic stresses showed that many genes were associated with cell wall metabolism; for example, a cellulose synthase (*PME3*) and a UDP-D-xylose 4-epimerase (*MUR4*) were repressed (Zhu et al., 2013). In the present study, based on transcriptome data, several candidate genes were selected for further experiments. The qRT-PCR results showed that the expression of *GhGT47_94* and *GhGT47_105* changed significantly during cold treatment, potentially improving the tolerance of cotton to cold (**Figure 10**).

The plant immune response is activated by wall-degrading molecules, which are produced when phytopathogenic fungi break down the integrity of the cell wall (Pogorelko et al., 2014). Pectin is critical for immunity in *A. thaliana* (Bethke et al., 2016). In the present study, the results of a cis-acting element analysis of the GhGT47 promoter regions revealed multiple cis-acting elements that responded to abiotic stress and pathogens. In addition, many cis-acting elements related to phytohormones have been identified, especially those related to MeJA, which is an important mechanism of plant wound and pathogen responses (Creelman et al., 1992). Based on these analyses and the currently known relationship between cell walls and plant resistance, it can be inferred that GT47 genes are involved in a variety of stress responses that enhance resistance in cotton.

## Conclusions

Genes of the GT47 family are critical for cell wall synthesis. In the present study, GT47 gene family members were identified in four cotton species. A comprehensive bioinformatics analysis was performed to reveal the sequence conservation, biological characteristics and evolutionary relationship of GT47 genes in *Gossypium spp*. The main objective of our study was to explore the phylogenetic relationships, collinearity, gene structures, expansion patterns and cis-acting elements of the GT47 family. Finally, we identified several GT47s related to fiber development and stress response. Although the functions of most GT47s were not clear, the systematic bioinformatics analysis provided data support for the chromosome distribution, biological characteristics and expression patterns of the GT47 family in cotton as well as candidate genes for later functional studies using the qRT-PCR experiment.

## Data Availability

All datasets generated for this study are included in the manuscript and the **Supplementary Files**.

## Author Contributions

HTW and AW conceived and designed the study and prepared the manuscript. AW, PH, HLW, HS, and SC performed the experiments. PC, QM, LG, and MZ assisted with the analysis and interpretation of the data. SY participated in the design of the experiments and provided a critical review. All authors have read, edited, and approved the current version of the manuscript.

## Funding

This work was supported by the National Natural Science Foundation of China (Grant 31621005) and the China Agriculture Research System (CARS-15-06).

## Conflict of Interest Statement

The authors declare that the research was conducted in the absence of any commercial or financial relationships that could be construed as a potential conflict of interest.

## Supplementary Material

Table S1 Primer pairs used in the qRT-PCR analysis.

Table S2 Gene locus information for GT47s in Arabidopsis thaliana, Populus trichocarpa, G. arboreum, G. raimondii, G. hirsutum and G. barbadense. Gene location information was obtained from gene annotation files, and the locations of exostosin domains were determined using the online Simple Modular Architecture Research Tool (SMART).

Table S3 Duplicate types of GT47s in G. hirsutum and G. barbadense. Dispersed means that the gene might arise from transposition; WGD or segmental means that the gene might arise from whole-genome duplication or segmental duplication; and tandem means that the gene might arise from tandem duplication and insertion of some other genes.

Table S4 The Ka/Ks ratios of duplicated gene pairs between the A and D subgenomes of allotetraploid cottons and their corresponding ancestral A and D diploid genomes, respectively. The homologous gene pairs were identified by the results of BLAST and collinearity analysis. When both Ka and Ks were equal to 0, Ka /Ks was considered equal to 1. When only Ks was equal to 0, it was marked as Ka>>Ks.

Table S5 Detailed physicochemical characteristics of GT47 proteins of A. thaliana, P. trichocarpa, G. arboreum, G. raimondii, G. hirsutum and G. barbadense. The pI and MW were computed by the Compute pI/Mw tool. The N-terminal signal peptide and transmembrane helices were predicted by the SignalP 4.1 server and the TMHMM v.2.0 server, respectively. Subcellular localizations of GT47 proteins were predicted by DeepLoc.

Table S6 Statistical results of stress response cis-acting elements in the promoter segments of GT47s. These cis-acting elements were identified using PlantCARE software with the upstream 2000-bp sequences of cotton GT47s.
