# Supplementary figures and images for: Genome-Wide Identification and Characterization of Glycosyltransferase Family 47 in Cotton
Source: Front Genet. 2019 Sep 11;10:824. doi: 10.3389/fgene.2019.00824 (PMC6749837; doi:10.3389/fgene.2019.00824)

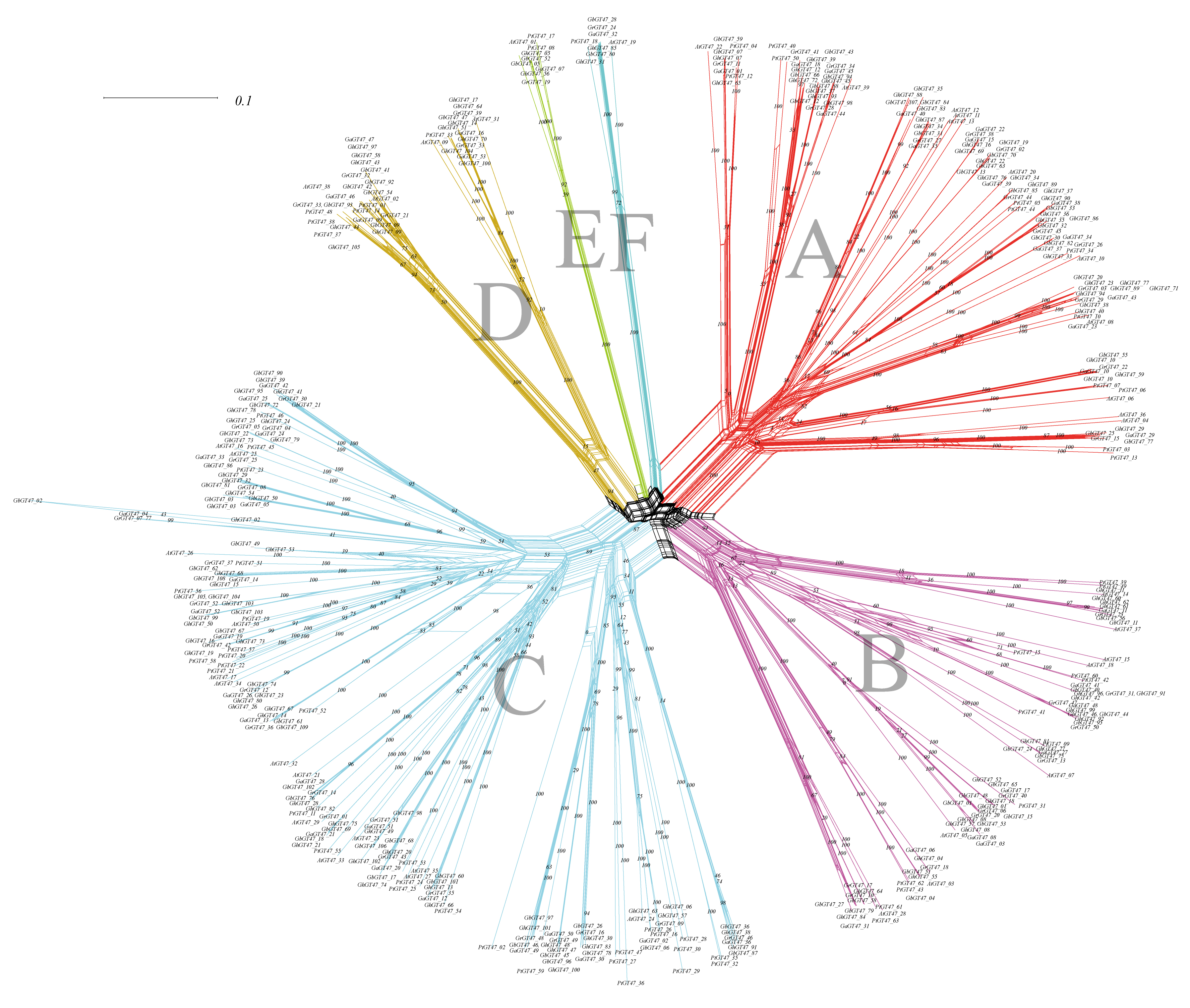

Supplement: Supplementary file 1 [file Image_1.jpeg]

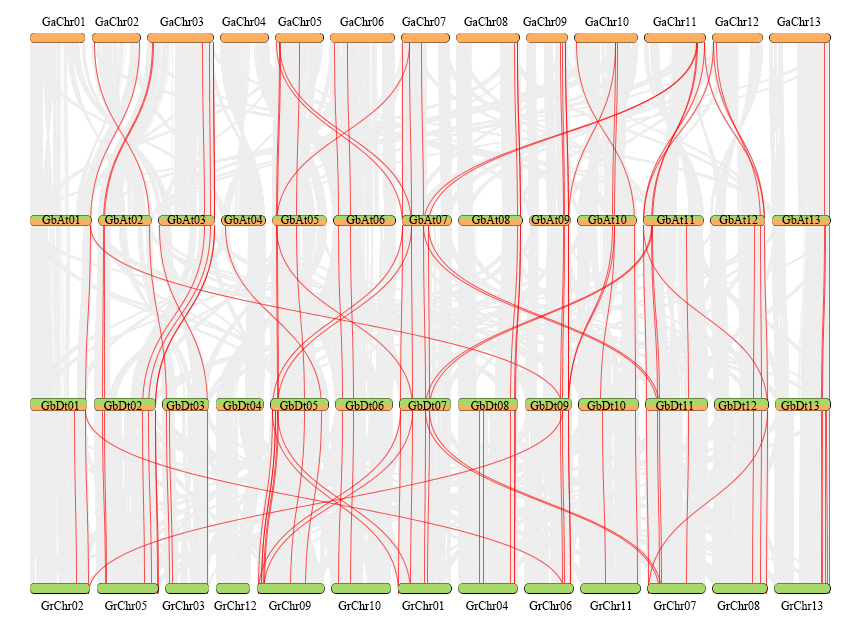

Supplement: Supplementary file 2 [file Image_2.jpeg]

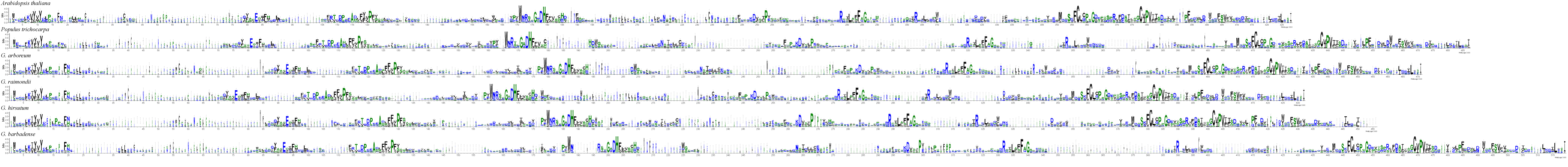

Supplement: Supplementary file 3 [file Image_3.jpeg]

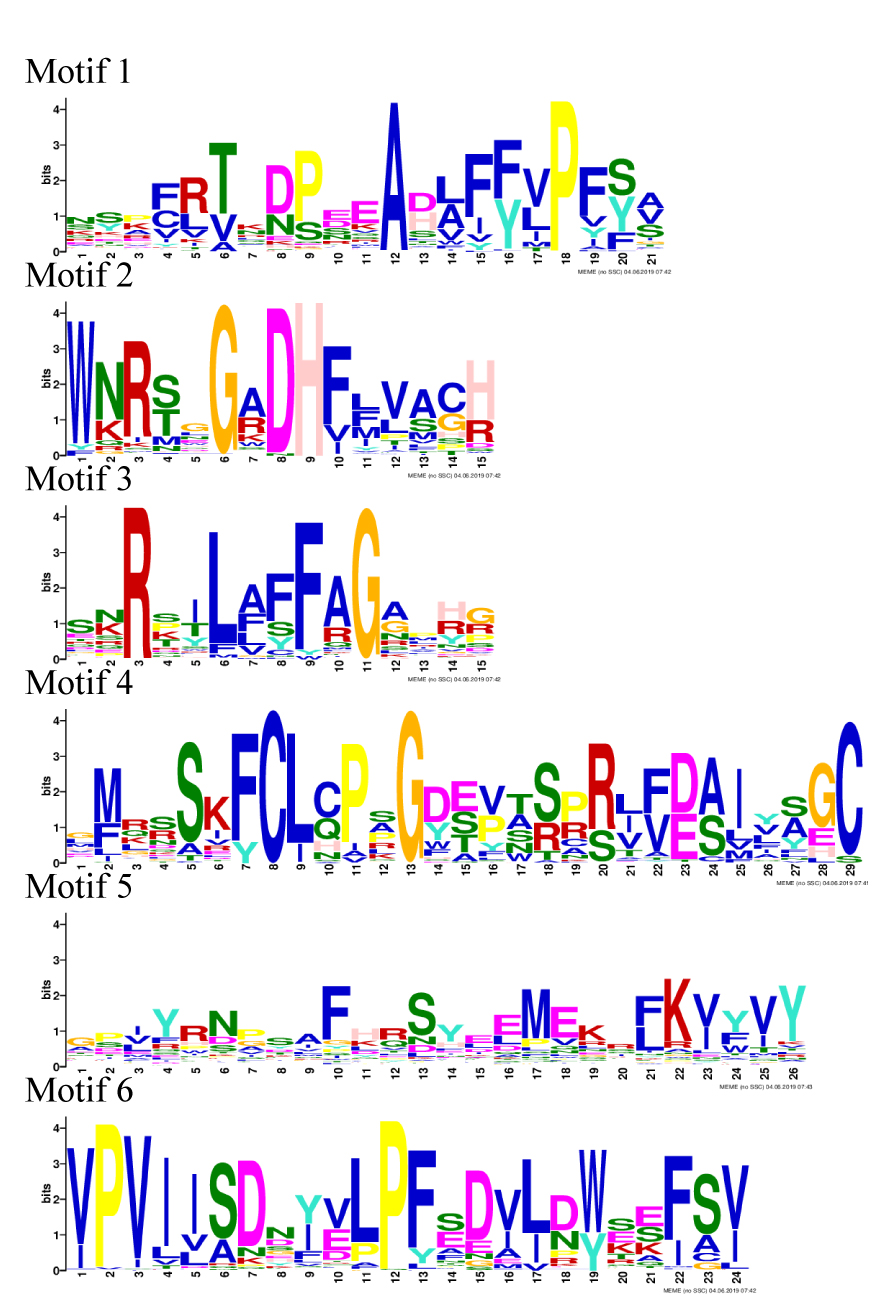

Supplement: Supplementary file 4 [file Image_4.jpeg]
