# Supplementary material for: Genome-Wide Identification and Characterization of Glycosyltransferase Family 47 in Cotton
Source: Front Genet. 2019 Sep 11;10:824. doi: 10.3389/fgene.2019.00824 (PMC6749837; doi:10.3389/fgene.2019.00824)
Supplement: Supplementary file 5 [file Image_5.pdf]

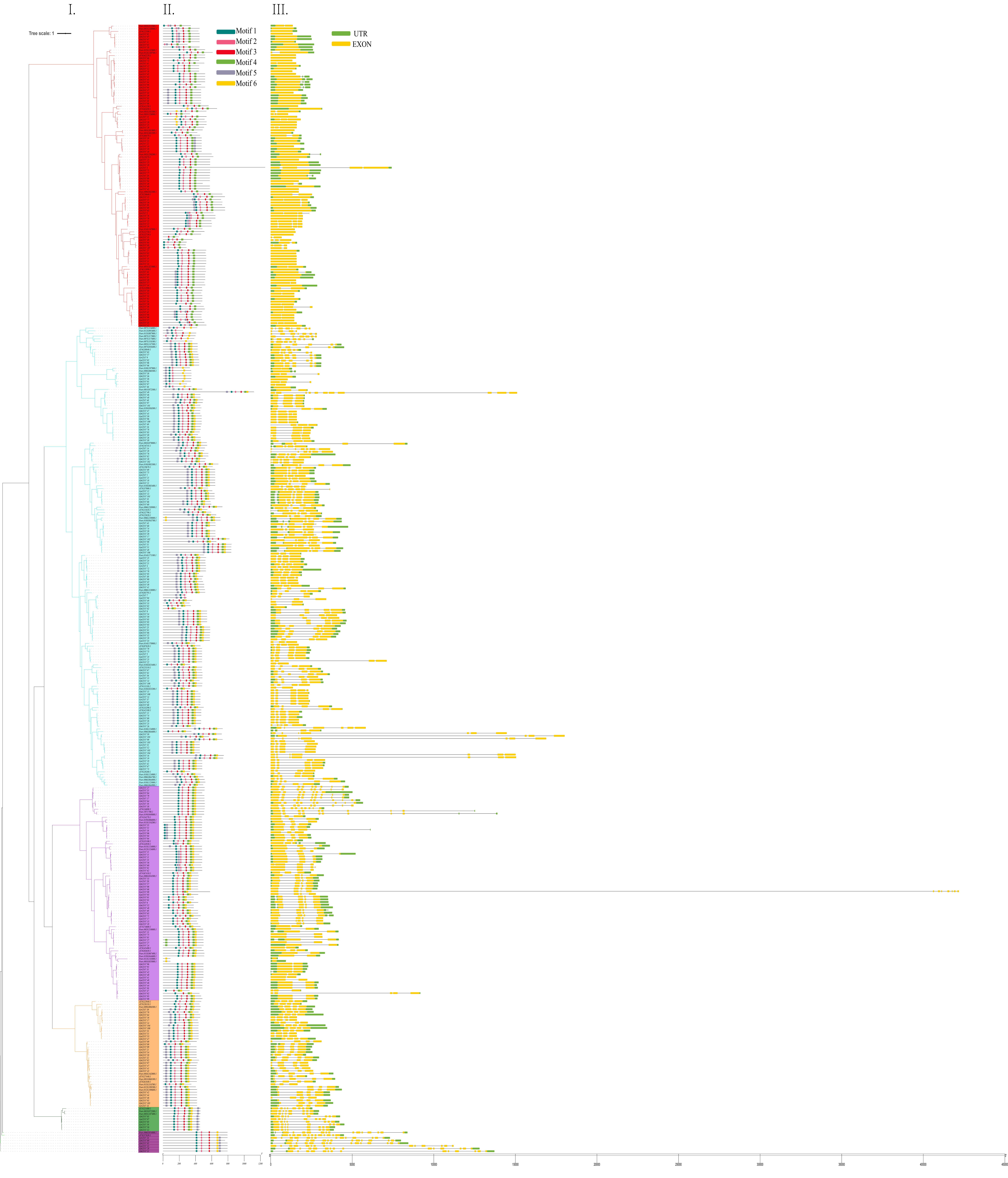
I.
II.
III.
Tree scale: 1
Motif 1
Motif 2
Motif 3
Motif 4
Motif 5
Motif 6
UTR
EXON
